# Mapping Eight Decades of Vaccination Social Science: Bibliometric Analysis of Global Research Trends

**DOI:** 10.3390/vaccines13111138

**Published:** 2025-11-04

**Authors:** Chinwe Iwu-Jaja, Oluwatosin Nkereuwem, Chidozie D. Iwu, Akhona V. Mazingisa, Anelisa Jaca, Duduzile Ndwandwe, Charles S. Wiysonge

**Affiliations:** 1 World Health Organization Regional Office for Africa, Brazzaville P.O. Box 06, Congo; charles.wiysonge@mrc.ac.za; 2 Medical Research Council Unit, London School of Hygiene & Tropical Medicine, Banjul P.O. Box 273, The Gambia; oluwatosin.nkereuwem@lshtm.ac.uk; 3 School of Health Systems and Public Health, Faculty of Health Sciences, University of Pretoria, Pretoria 0028, South Africa; chidoziedeclan@gmail.com; 4 Department of Epidemiology, School of Public Health, University of Washington, Seattle, WA 98195, USA; 5 Department of Community Health Studies, Faculty of Health Sciences, Durban University of Technology, P.O. Box 1334, Durban 4000, South Africa; akhonamazingisa04@gmail.com; 6 Cochrane South Africa, South African Medical Research Council, P.O. Box 19070, Cape Town 7505, South Africa; anelisa.jaca@mrc.ac.za (A.J.); duduzile.ndwandwe@mrc.ac.za (D.N.)

**Keywords:** bibliometric analysis, vaccination, social science, vaccine hesitancy, global health, research trends, COVID-19, health equity

## Abstract

Background: Despite growing recognition of vaccination social science as essential to immunization strategies, the field’s evolution, geographic distribution, and research patterns remain poorly characterized. This study provides the first comprehensive mapping of the social science literature on vaccination over eight decades. Methods: We conducted a bibliometric analysis of peer-reviewed publications indexed in PubMed from their inception, using a systematic search strategy that combined vaccination and social science terms. Publications were analyzed using the Bibliometrix R package (version 5.0) to examine temporal trends, author productivity, institutional contributions, geographic distribution, and thematic evolution globally. Results: We retrieved 8005 eligible publications. Analysis highlighted three chronological research phases: sporadic early work (1945–1980, *n* = 85), sustained growth (1981–2019, *n* = 2743), and unprecedented expansion since the COVID-19 era (2020–2024, *n* = 4563). Annual publications reached a peak in 2022 (*n* = 1686). Research spans 146 countries but remains concentrated in high-income countries, with the United States (*n* = 10,230), China (*n* = 3796), and Canada (*n* = 2288) leading production. The top 20 institutions were from the United States (*n* = 8), United Kingdom (*n* = 4), and Canada (*n* = 3), with a few institutions from African countries. International collaboration was moderate (19.44%). Thematic analysis revealed a clear evolution from biological science (1963–1999) to socio-behavioural science, with an emphasis on vaccine hesitancy, trust, communication, and health equity (2015–2024). Conclusions: Vaccination social science has grown steadily over the decades, with a sharp rise in research during the COVID-19 pandemic. Most studies were from high-income countries, underscoring the need for enhanced social science capacity in low- and middle-income countries. As the focus of immunization efforts shifts toward issues like vaccine hesitancy and trust, broader collaboration and inclusion will be key to improving vaccine uptake worldwide.

## 1. Introduction

Vaccination has long been recognized as one of the most cost-effective public health interventions, significantly reducing morbidity and mortality associated with infectious diseases [1,2]. Since the launch of the Expanded Programme on Immunization (EPI) 50 years ago, vaccines have averted an estimated 154 million deaths and contributed to a 40% decline in infant mortality worldwide [1].

However, the success of vaccination programmes is not determined by biomedical efficacy alone. Uptake of efficacious vaccines is indispensable for public health impact. Growing evidence highlights that social and behavioural factors play a central role in shaping trust in, community engagement for, and uptake of vaccines. Social dimensions, ranging from cultural norms, political ideologies, and religious beliefs to communication patterns and institutional trust, often determine the real-world effectiveness of immunization strategies [3,4,5]. This convergence of social, behavioural, and political determinants defines what we refer to in this paper as the field of vaccination social science, an interdisciplinary domain encompassing psychology, sociology, anthropology, behavioural economics, risk communication, and political science [6,7]. Although the term has not been formally standardized in the literature, it is used here operationally to describe research that applies social and behavioural science perspectives to understand and address human, societal, and institutional factors influencing vaccine confidence, trust, and uptake.

Historically, vaccination efforts have long encountered resistance rooted in sociopolitical dynamics. In the 19th and early 20th centuries, smallpox vaccination campaigns were met with backlash due to fears of compulsory medical intervention, religious objections, and safety concerns [8].

Similarly, polio eradication efforts in the early 2000s were severely hampered in northern Nigeria, where religious and political leaders disseminated rumours that the oral polio vaccine caused infertility, leading to widespread refusals and disease resurgence [9]. These challenges have catalyzed a surge in social science research, particularly since the COVID-19 pandemic, which underscored how misinformation, political polarization, and institutional distrust can undermine public confidence in vaccines. The World Health Organization in 2019 declared vaccine hesitancy as one of the top ten threats to global health, elevating the urgency of research into the social dimensions of vaccination [3].

During the pandemic, the pace of vaccine development outstripped public understanding, giving rise to widespread hesitancy and conspiracy theories, which could not be addressed by biomedical solutions alone [10,11,12]. This therefore reinforces the critical importance of integrating behavioural insights, community engagement, and participatory communication strategies into immunization efforts [13,14]. Studies emphasize that understanding public sentiments, social trust, and behavioural drivers through methods grounded in behavioural science, risk communication, and participatory research can help design more responsive, equitable, and effective vaccination strategies [15].

The field of vaccination social science has thus expanded in both scope and relevance, providing tools not only to diagnose the roots of vaccine hesitancy but also to guide programmatic adaptations during health emergencies and routine immunization campaigns alike [16].

Despite its growing importance, the structure and evolution of vaccination social science literature remain poorly understood. To the best of our knowledge, no comprehensive bibliometric study has mapped the global knowledge production in this field, its historical growth patterns, key contributors, institutional drivers, and geographical disparities. Bibliometric analysis, a quantitative method for evaluating the scientific literature, provides critical insights into research trends, productivity, collaboration networks, and thematic shifts over time [17], serving as a foundational tool for advancing scientific knowledge and guiding evidence-informed research and policy decisions.

The objective of this study was to assess temporal publication trends, prolific authors and institutions, geographic patterns, and emerging thematic areas in vaccination social science research. The findings offer a historical lens through which the evolution of social science perspectives on vaccination can be examined and inform future investments and policy focus, and any future work related to its taxonomy.

## 2. Materials and Methods

### Data Source and Search Strategy

A bibliometric analysis of publications related to vaccine social sciences was conducted based on data retrieved from the PubMed database, covering all records from inception to 8 June 2025. The PubMed database provides extensive coverage of health-related articles with integrated bibliometric information [18]. The final search query was constructed by combining two sets of terms using relevant Boolean operators: Vaccine-related terms (e.g., vaccine, vaccination, immunisation, immunization) and social science-related terms (e.g., sociology, anthropology, psychology, communication, behavioural science, vaccine hesitancy, public trust, community participation). The complete search syntax is provided in Supplementary Table S1.

The search was limited to titles to ensure retrieval of publications with an explicit focus on vaccination and social science concepts. No restrictions were applied to document type, study design, or language at the search stage. The search process was iterative- refinements to the search string were made after initial testing to improve precision and remove irrelevant records. To enhance reliability, a second reviewer independently verified the retrieved dataset to ensure that the included records were relevant to the study scope. We did not apply additional inclusion or exclusion criteria, as the purpose of the study was to provide a comprehensive overview of the entire body of vaccination social science literature indexed in PubMed.

Following the search, the articles were retrieved, converted into a bibliographic data frame, and analyzed using the Bibliometrix R package [17,19]. As a first step, the publications were summarized into the number of articles retrieved, types (whether journals, books, etc.), article average age, and authors who published during the study period. Additionally, we determined the average number of co-authors per article [17].

Annual growth rate (AGR) values were automatically computed using the Bibliometrix R package. Next, we determined the annual scientific productions, which is the number of articles published annually within the study duration. Then we compiled the list of the top prolific authors and institutions, top productive countries, the geographical distribution of country productivity, and an analysis of trend topics. International collaboration was assessed using the Multiple-Country Publications (MCP) metric generated by Bibliometrix, based on distinct author affiliations listed per publication. Publications with authors from institutions in more than one country were classified as MCPs, while those with authors from a single-country affiliation were classified as Single-Country Publications (SCPs) [17].

## 3. Results

### 3.1. Overview

A total of 8005 records were retrieved and included in the analysis, with the earliest study dating back to 1945. Table 1 provides a summary of key bibliometric indicators, including the number of sources, authorship volume, annual growth rate, and extent of international collaboration.

### 3.2. Publication Trends over Time

**Figure 1:**
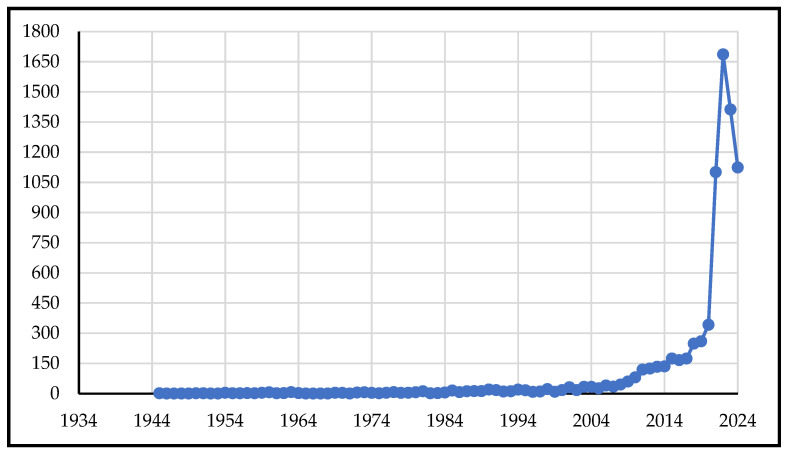
Annual publication trends in vaccination social science research, 1945–2024.

The temporal distribution of vaccination social science publications from 1945 to 2024, as shown in Figure 1, revealed three distinct phases of research activity. The earliest publication in the dataset appeared in 1945, followed by sporadic output until 1980, resulting in a total of 85 publications over the 35-year period. Research activity remained minimal and inconsistent, with several years producing zero publications (1946–1949, 1952–1953, 1967–1968). However, a sustained increase in publication volume began in 1981, with annual output growing from 11 publications in 1981 to 260 in 2019. During these 39 years (1981–2019), a total of 2743 publications were produced, representing an average annual growth rate of approximately 8.9%. The decade 2011–2021 demonstrated a notable trajectory of increasing research activity. There were moderate increases from 2011 to 2014 (119 to 135 publications), followed by a notable acceleration in 2015 (174 publications, 28.9% increase). The transition period 2019–2021 marked a dramatic shift in publication outputs. The period 2020–2024 demonstrated an unprecedented increase in publication volume from 342 to 1124 publications per year, with a peak in 2022, reaching 1686 publications.

### 3.3. Institutional Distribution of Publications

**Figure 2:**
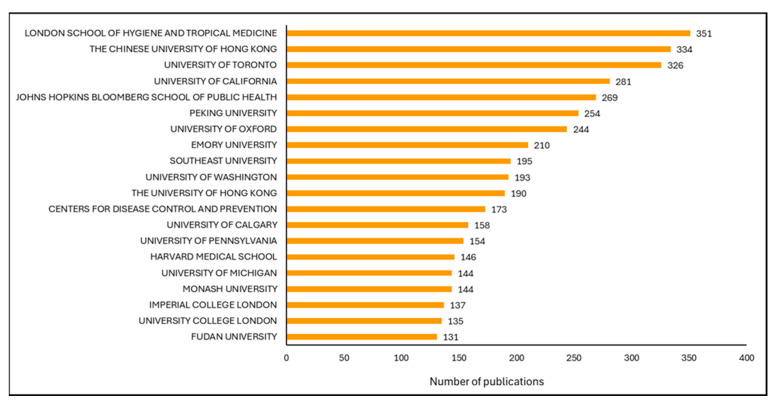
Top 20 most productive institutions in vaccination social science research by publication counts.

The top 20 most productive institutions contributed between 131 and 351 publications each (Figure 2). The London School of Hygiene and Tropical Medicine led with 351 publications, followed by the Chinese University of Hong Kong (334) and the University of Toronto (326). The top five most prolific institutions also include the University of California (281) and Johns Hopkins Bloomberg School of Public Health (269).

Among the top 20 institutions, eight were located in the United States of America (USA), four in the United Kingdom of Great Britain and Northern Ireland (UK), three in Canada, two in Hong Kong, two in China, and one in Australia. Government agencies were represented in the top 20 by the USA Centers for Disease Control and Prevention (173 publications, ranked 12th). The complete ranking of the top 50 institutions is presented in Supplementary Table S2, with publication counts ranging from 351 to 86 publications.

### 3.4. Geographical Distribution of Publications on Vaccination Social Science

**Figure 3:**
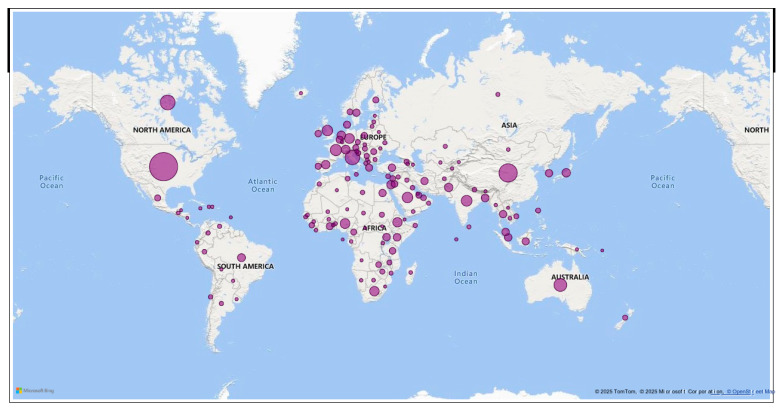
Geographical distribution of publications on vaccination social science *. * *The size of the cycle represents the number of publications*.

**Figure 4:**
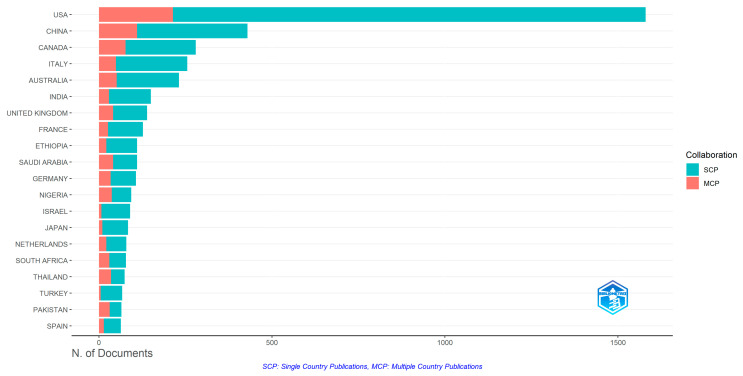
Top 20 countries by publication output and collaboration pattern.

Research activity spans across 146 countries globally (Figure 3), with disparities in research productivity. The top 20 publishing countries represent five UN regional groups according to the United Nations Statistics Division (UNSD) geographic classification [20], which provides a standardized framework for comparing countries and regions globally: Northern America (USA, Canada), Europe (Italy, UK, France, Germany, Netherlands, Spain), Asia (China, India, Japan, Thailand, Pakistan, Saudi Arabia, Turkey, Israel), Africa (Ethiopia, Nigeria, South Africa), Western European and other states (USA, Canada, Israel) and Oceania (Australia). The top 5 highest countries were the USA, China, Canada, Italy, and Australia. Three African countries feature among the top 20, namely, Ethiopia (9th), Nigeria (12th), and South Africa (16th). Most of these countries demonstrated a greater number of substantial single-country publications (SCPs) compared to multiple-country publications (MCPs) (Figure 4).

### 3.5. Author Productivity Analysis

**Figure 5:**
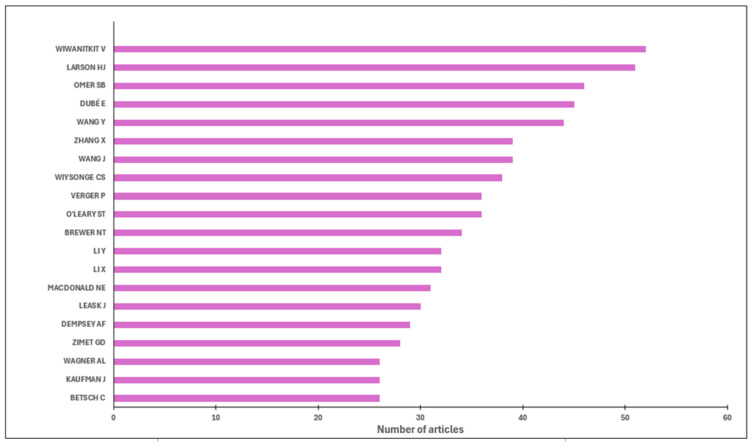
Top prolific authors for vaccination social science based on results from the PubMed database only.

The top 20 most prolific authors are shown in Figure 5. Wiwanitkit V, Larson HJ, Omer SB, Dubé E, Wang Y, Wang J were notable authors from this list leading vaccination social science research.

### 3.6. Shifting Thematic Priorities in Vaccination Social Science Across Three Eras

Trend topic analysis using Bibliometrix revealed clear shifts in thematic focus over the study period. It is noteworthy that trend topic analysis identified relevant terms only from 1963 onwards. These inflection points informed the division of the timeline into three eras: 1963–1999, 2000–2014, and 2015–2024. Segmenting the analysis in this way allowed us to better highlight the evolution of research priorities within vaccination social science over time (Supplementary Figures S1–S3). This evolution of vaccination social science research reveals distinct thematic shifts across the three time periods. Between 1963 and 1999, early research focused predominantly on immunological concepts such as “vaccinia virus,” “antibody formation,” and “bacterial vaccines,” with emergent themes linked to health services and hospital personnel psychology. The 2000–2014 period showed expansion into population-level topics including “risk factors,” “health promotion,” and “data collection,” alongside a notable rise in studies on “immunization programs,” “urban populations,” and “rural health.” Finally, between 2015 and 2024, the landscape shifted dramatically in response to COVID-19, with dominant themes including “vaccination hesitancy,” “COVID-19 vaccines,” “health belief model,” and “socioeconomic factors.” The period began at a time when conversations on vaccine hesitancy were gaining ground with the publication in 2015 of the report of the Strategic Advisory Group of Experts on Immunization (SAGE) Working Group on Vaccine Hesitancy [20]. This period reflects a robust integration of behavioural models, demographic factors (e.g., age, gender), and health equity concerns. The progression illustrates a clear movement from biologically focused research toward socio-behavioural and systems-level inquiry, highlighting the maturing complexity and public health relevance of the field.

## 4. Discussion

This bibliometric analysis provides the first comprehensive mapping of vaccination social science literature, spanning 80 years, highlighting a dynamic, expanding, and increasingly policy-relevant research landscape. With 8005 publications analyzed from 1945 to 2024, the findings illustrate not only the exponential growth in output, especially post-2000, but also the maturation of the field from basic psychosocial inquiries to complex, interdisciplinary investigations addressing vaccine hesitancy, trust, communication, and equity.

The increasing volume of vaccination social science publications aligns with broader recognition of the critical role non-biomedical factors play in immunization outcomes. From smallpox-era resistance to modern vaccine scepticism, the uptake and success of vaccination programs have consistently hinged on public perception, trust, and behaviour, not just product availability or efficacy [9]. As reflected in WHO’s designation of vaccine hesitancy as a top ten global health threat in 2019 [3], the field has transitioned from a niche domain to a vital scientific pillar underpinning immunization strategy and policy.

The COVID-19 pandemic provided a particularly powerful inflection point. Our analysis shows a dramatic surge in publication volume during the 2020–2024 period, with 2022 marking the peak. This coincides with a global reckoning on the limits of biomedical communication alone. Misinformation, political polarization, and widespread distrust rendered many COVID-19 vaccine rollouts vulnerable to delays, refusals, and inequities [11,21,22,23,24,25,26,27,28]. In this context, the demand for insights from psychology, anthropology, sociology, and behavioural economics accelerated, validating the centrality of social science perspectives in vaccine discourse.

Our trend topic analysis spanning three time periods (1963–1999, 2000–2014, and 2015–2024) illustrates a clear evolution in research priorities. Early publications largely emphasized immunological constructs such as “vaccinia virus” and “antibody formation.” Over time, this biologically anchored focus gave way to population-level health promotion, data collection, and urban/rural disparities, culminating most recently in a robust preoccupation with vaccine hesitancy, behavioural theory (e.g., health belief model), and equity concerns. This thematic trajectory affirms the maturation of vaccination social science into a systems-level field that interrogates both individual beliefs and institutional structures.

Notably, the prominence of terms such as “vaccine hesitancy,” “COVID-19 vaccines,” “risk communication,” and “public trust” in the most recent period reflects the deep entanglement of immunization with digital, political, and sociocultural environments. This finding aligns with other research suggesting that resistance to vaccines is no longer merely a function of knowledge deficits but is often rooted in values, identity, and institutional legitimacy [5,6,29]. Despite global participation across 146 countries, our findings highlight a highly uneven distribution of research productivity. The most prolific institutions, including the London School of Hygiene and Tropical Medicine, University of Toronto, and Johns Hopkins Bloomberg School of Public Health, are in high-income countries, with eight of the top 20 institutions based in the United States. While these hubs bring extensive expertise and capacity, their dominance also raises concerns about the underrepresentation of low- and middle-income country perspectives, particularly from regions where vaccination challenges are most acute.

Indeed, only a handful of African institutions, such as the University of Cape Town, the South African Medical Research Council, and the University of Gondar, appear among the top 50. This limited visibility may reflect persistent inequities in research funding, infrastructure, and authorship, which are well documented in global health [30,31,32]. Moreover, much of the social science literature on vaccination in low- and middle-income countries continues to be led or co-led by researchers based in, raising questions about agenda setting, local ownership, and contextual relevance [33,34].

The geographic skew is further compounded by a relatively low rate of international co-authorship (19.44%), suggesting that transnational collaboration remains underdeveloped in vaccination social science. In a field so deeply shaped by cultural context, participatory research and equitable partnerships are not only ethically desirable but scientifically necessary. It is important to note, however, that our estimation of international collaboration is based on multiple-country publications (MCPs) derived from author affiliations indexed in PubMed. This metric, while widely used in bibliometric analysis, may underestimate the true extent of transnational collaboration, particularly where global South researchers are affiliated with institutions in high-income countries, or where informal or undocumented partnerships exist. As such, our findings should be interpreted as indicative of formal institutional collaboration, rather than exhaustive of all collaborative dynamics.

### Implications for Policy and Research

Taken together, our findings highlight both the achievements and unfinished agenda of vaccination social science. The growth in volume and thematic diversity signals increasing integration into global health discourse. However, important gaps remain, including geographic imbalances, limited South-led research, and underuse of social science approaches in routine immunization systems.

To unlock the transformative potential suggested by this evolving body of work, the field must now consolidate its identity; articulating shared definitions, establishing a practical taxonomy, and embedding vaccination social science more systematically into institutional structures. These actions should be grounded in equity, with intentional efforts to amplify low- and middle-income country participation and leadership. Similar calls have been made in adjacent domains of vaccine access and implementation, emphasizing the need to rebalance global health architecture in ways that are just, inclusive, and co-led by the Global South [35].

## 5. Limitations

This study represents a preliminary effort to map the landscape of vaccination social science literature using bibliometric methods. While the scale and breadth of the dataset offer important insights, several limitations should be acknowledged. First, although we explored the potential for disciplinary classification (e.g., into psychology, anthropology, communication, and political science), the method based on automated rule-based natural language processing did not yield consistently reliable results. We therefore chose not to include disciplinary breakdowns in the final analysis. Future research may benefit from integrating more robust approaches, such as supervised machine learning, manual coding, and expert consensus methods, to develop a practical and reproducible taxonomy of vaccination social science.

Second, our reliance on PubMed as the sole data source may have excluded relevant articles indexed in other interdisciplinary or region-specific databases. Nonetheless, PubMed offers extensive coverage [18] and indexes a broad range of journals that regularly publish on vaccination-related social science, including Vaccine, Social Science & Medicine, Human Vaccines & Immunotherapeutics, BMC Public Health, and others covering behavioural, policy, equity, and communication perspectives. Our selection was based on the technical compatibility with bibliometric tools and the ability to process large volumes of data efficiently. Broader database inclusion in future studies could enhance the comprehensiveness and diversity of results.

Lastly, this bibliometric methodology has its own limitations. The analysis did not allow for detailed classification of studies by research type or clear categorization of subject areas [36], especially within the vaccination social science spectrum. Future analyses that incorporate study-type classification, possibly supported by supervised machine learning or manual coding, could provide a more refined understanding of research emphasis and existing gaps [36]. Additionally, while international collaboration was assessed, this study did not examine patterns of authorship leadership (e.g., first or senior author positions), which could offer deeper insights into research ownership and capacity development, particularly across low- and middle-income countries [36].

## 6. Conclusions

This bibliometric analysis demonstrates that vaccination social science has evolved from an emerging but previously overlooked field to a core element of global immunization strategies, with COVID-19 marking a major inflection point in research growth and interdisciplinary engagement. However, research output remains disproportionately concentrated in high-income countries, limiting representation from regions most affected by vaccine-preventable diseases. As the world continues to address immunization gaps and prepare for future pandemics, advancing vaccination social science will require more equitable participation and locally relevant perspectives. Sustained investment in this field can strengthen public trust, improve programme implementation, and ensure that vaccination fulfils its promise as one of the most powerful tools for global public health.

## Figures and Tables

**Table 1:** Overview of publication volume, authorship, and international collaboration in vaccination social science research.

Description	Results
Number of articles	8005
Sources (Journals, Books, etc.)	1466
Overall annual growth rate (percentage)	2%
Total number of authors	33,026
International co-authorships (percentage)	19.44%

## Data Availability

Data generated for this review were all from publicly available publications.

## Supplementary Materials

The following supporting information can be downloaded at: https://www.mdpi.com/article/10.3390/vaccines13111138/s1, Figure S1: Trend topics between 1963–1999; Figure S2: Trend topics between 2000–2014; Figure S3: Trend topics between 2015–2024; Table S1: Search strategy for vaccination social science publications on PubMed from 1945 to 2024 (Search conducted on 9 June 2025); Table S2: Top 50 institutions by publication count in vaccination social science research.
