# Supplementary material for: Mapping Eight Decades of Vaccination Social Science: Bibliometric Analysis of Global Research Trends
Source: Vaccines (Basel). 2025 Nov 4;13(11):1138. doi: 10.3390/vaccines13111138 (PMC12656872; doi:10.3390/vaccines13111138)
Supplement: Supplementary file 1 [file vaccines-13-01138-s001.zip › vaccines-3884828-supplementary.pdf]

Appendix

Supplementary Table S1: Search strategy for vaccination social science publications on PubMed from 1945 to 2024 (Search conducted on 9th June 2025).

Search	Query	Results
#3	Search: #1 AND #2 **from 1000/1/1 - 2024/12/31**	8,005
#2	Search: social science*[Title] OR sociolog*[Title] OR anthropolog*[Title] OR ethnograph*[Title] OR psycholog*[Title] OR communication*[Title] OR "risk communication"[Title] OR "health communication"[Title] OR "knowledge attitude* practice"[Title] OR "community engagement"[Title] OR "community participation"[Title] OR "public trust"[Title] OR "vaccine hesitancy"[Title] OR acceptan*[Title] OR uptake[Title] OR "behavioral science*"[Title] OR "behavioural science*"[Title] OR "behavioral change"[Title] OR "behavioural change"[Title] Sort by: Most Recent	289,105
#1	Search: Vaccine[Title] OR vaccination[Title] OR immunisation[Title] OR immunization[Title] OR vaccin*[Title] Sort by: Most Recent	273,749

Supplementary Table S2: Top 50 institutions by publication count in vaccination social science research from 1945 to 2024 (Search conducted on 9th June 2025).

**Affiliation**	**Articles**
LONDON SCHOOL OF HYGIENE AND TROPICAL MEDICINE	351
THE CHINESE UNIVERSITY OF HONG KONG	334
UNIVERSITY OF TORONTO	326
UNIVERSITY OF CALIFORNIA	281
JOHNS HOPKINS BLOOMBERG SCHOOL OF PUBLIC HEALTH	269
PEKING UNIVERSITY	254
UNIVERSITY OF OXFORD	244
EMORY UNIVERSITY	210
SOUTHEAST UNIVERSITY	195
UNIVERSITY OF WASHINGTON	193
THE UNIVERSITY OF HONG KONG	190
CENTERS FOR DISEASE CONTROL AND PREVENTION	173
UNIVERSITY OF CALGARY	158
UNIVERSITY OF PENNSYLVANIA	154
HARVARD MEDICAL SCHOOL	146
MONASH UNIVERSITY	144
UNIVERSITY OF MICHIGAN	144

IMPERIAL COLLEGE LONDON	137
UNIVERSITY COLLEGE LONDON	135
FUDAN UNIVERSITY	131
UNIVERSITY OF CAPE TOWN	126
UNIVERSITY OF OTTAWA	126
CHAN SCHOOL OF PUBLIC HEALTH	122
DALHOUSIE UNIVERSITY	122
KING'S COLLEGE LONDON	122
SOUTH AFRICAN MEDICAL RESEARCH COUNCIL	120
UNIVERSITY OF NORTH CAROLINA	119
NATIONAL CENTER FOR IMMUNIZATION AND RESPIRATORY DISEASES	117
KING SAUD UNIVERSITY	115
UNIVERSITY OF BRITISH COLUMBIA	112
UNIVERSITY OF SYDNEY	110
UNIVERSITY OF COLORADO ANSCHUTZ MEDICAL CAMPUS	107
MINISTRY OF HEALTH	101
BAYLOR COLLEGE OF MEDICINE	98
SWANSEA UNIVERSITY MEDICAL SCHOOL	96
UNIVERSITY OF BRISTOL	95
UNIVERSITY OF GONDAR	95
MCGILL UNIVERSITY	94
JOHNS HOPKINS UNIVERSITY	93
UNIVERSITY OF ARKANSAS FOR MEDICAL SCIENCES	92
UNIVERSITY OF NEW SOUTH WALES	92
ZHENGZHOU UNIVERSITY	92
CHINESE ACADEMY OF MEDICAL SCIENCES AND PEKING UNION MEDICAL COLLEGE	90
UNIVERSITY OF ALBERTA	90
UNIVERSITY OF FLORENCE	90
CITY UNIVERSITY OF NEW YORK (CUNY)	88
SUN YAT-SEN UNIVERSITY	86
UNIVERSITY OF LONDON	86
UNIVERSITY OF NORTH CAROLINA AT CHAPEL HILL	86
YALE UNIVERSITY	86

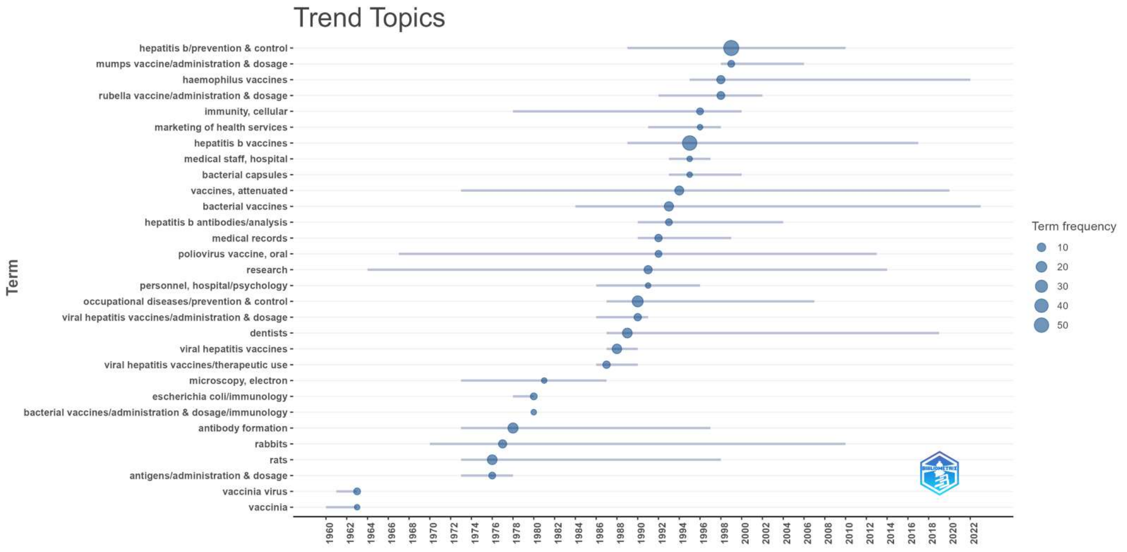

Supplementary Figure S1: Trend topics between 1963-1999.

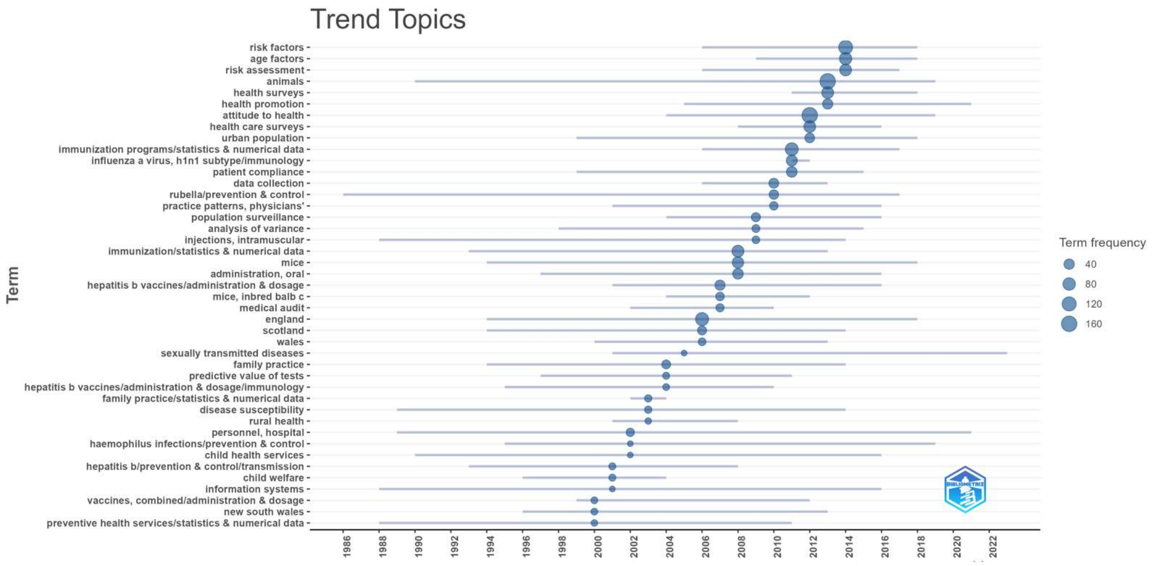

Supplementary Figure S2: Trend topics between 2000-2014.

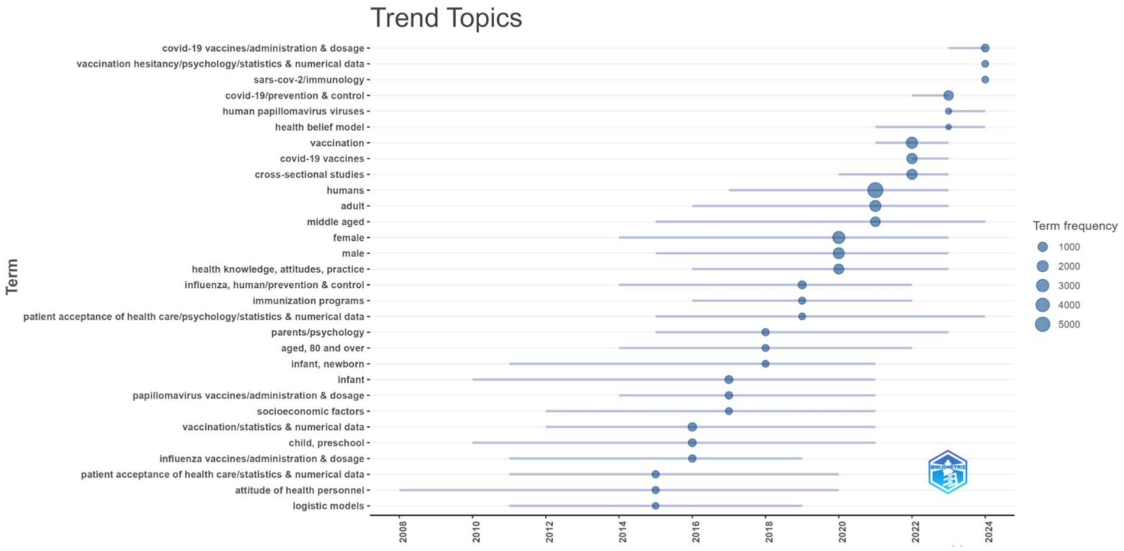

Supplementary Figure S3: Trend topics between 2015-2024.
